# An segformer-mix vision transformer-based GIST segmentation in fused CT-PET images

**DOI:** 10.1371/journal.pone.0355109

**Published:** 2026-08-19

**Authors:** Venu Allapakam, Yepuganti Karuna

**Affiliations:** 1 Research Scholar, School of Electronics Engineering (SENSE), Vellore Institute of Technology, Vellore, India; 2 Associate Professor (Sr Grade -1), School of Electronics Engineering, VIT-AP University, Beside AP Secretariat, Amaravati, Andhra Pradesh, India; Chongqing Medical University, CHINA

## Abstract

Accurate segmentation of gastrointestinal stromal tumour (GIST) is always challenging task due to tissues intestines may have similar intensity values, which makes boundary delineation harder with only one source of contrast. Presently, clinicians make use of CT scans for manually annotate and diagnosis of GIST, which is ineffective and susceptible to subjectivity. To improve tumour delineation and effective segmentation of GIST, here we proposed two stage deep learning model for this purpose. In the first stage an DIF-Net is used to fuse CT and PET images, helps to improve quality of image for accurate delineation. In further stage the tumour region is effectively segmented by employing a Segformer architecture supplemented with a Mix Vision Transformer (MViT) encoder. The proposed methodology combines the powerful fusion characteristics of DIF-Net with the efficient segmentation characteristics of Segformer, allowing correctly capturing both local and global features, ultimately achieving superior segmentation results. The superiority of model performance is evaluated with multiple performance parameters for segmentation like mean Intersection Over Union (IoU), Pixel Accuracy, and dice coefficient, and for fusion it is PSNR (Peak Signal-to-Noise Ratio), SSIM (Structural Similarity Index), MI (Mutual Information), UIQI (Universal Image Quality Index) and Edge Preservation. Experimental results show that the proposed method achieves better segmentation and fusion quality than existing models, enhancing the accurate diagnosis.

## 1. Introduction

Gastrointestinal Stromal Tumour (GIST) is a rare type of tumour that originates in the digestive tract, most commonly in the stomach or small intestine [1]. Globally, gastrointestinal stromal Tumours (GISTs) have significant rates of occurrence and death [2]. In general, about 60–65% of this gist’s occur in the stomach, 20–25% in the small intestine, 10% in the colon and rectum, and 5% in the oesophagus. Determining treatment strategies and forecasting clinical stages mainly depend on the precise identification and segmentation of tumours region and the tissues around them [3,4].

Various imaging modalities are applied for detection, segmentation and follow up treatment of GISTs, such as the computerized tomography (CT), the magnetic resonance imaging (MRI), endoscopic ultrasound (EUS) and Positron Emission Tomography (PET). Due to its real-time nature, non-invasive and inexpensive CT, MRI and PET are used to assess patient specific gastrointestinal structure and function [5]. Computed Tomography (CT) and Positron Emission Tomography (PET) scans are among the most common imaging modalities used in medical diagnostics [6]. CT images, give us high-resolution anatomical information, while PET scans provide functional insight into tissue activity, which is essential for spotting abnormal metabolic activity, like in GISTs [7]. The main drawback in CT based examination alone lacks the contrast and functional detail to reliably segment GISTs, particularly when they have variable appearance and are in complex anatomical regions. Additionally, the issue with GIST segmentation using PET alone lack of anatomical precision due to low spatial resolution and absence of structural detail, which makes it unsuitable for fine segmentation without complementary modalities like CT or PET.

Traditional tumour region segmentation techniques used in clinical practice entail skilled physicians manually annotating and delineating abdominal CT scans. This combination of the two modalities CT-PET has been shown to be beneficial due to the ability to obtain both the high spatial resolution of the CT and the functional information provided by the PET, helps in improving the boundary delineation which is very harder with single image modality.

However, despite the combined benefits of CT-PET imaging, Accurate segmentation of anatomical structures is crucial in gastrointestinal stromal tumour (GIST) for diagnosing, determining treatment protocols. Segmenting relevant regions from the fused image data remains a complex task [8]. The process of identifying and extracting key anatomical structures, such as tumours or lesions, from fused CT-PET images is fraught with difficulties [9]. These challenges arise from the inherent differences in the resolution, contrast, and noise characteristics between the two imaging modalities. Additionally, the presence of low-contrast boundaries and the potential misalignment during image fusion can complicate the segmentation process further, reducing the accuracy of automated segmentation methods.

With the growing need for automated methodologies in clinical environments, where the speed and correctness of the process are crucial, the demand for reliable segmentation approaches is more indispensable than ever. Conventional segmentation methods like thresholding or region-growing methods often fail to capture structures of interest in fused CT-PET image [10]. Such techniques are often noisy sensitive and not enough to depict crucial information for prediction. Furthermore, the problem is more challenging due to the segmentation of large datasets within clinical settings, necessitating models that can generalize effectively across different patient types and disease stages.

As many researchers face these challenges, there is a lack of good scalable solutions to solve these problems leading to better segmentation. This has opened an exciting line of research using deep learning-based methods to improve segmentation performance [11,12]. However, improving delineation is the major limitation associated with the traditional fusion methods. Importantly, current models either do not translate well into real time clinical applications or have issues of the computational cost and/or scalability. Hence, to improve the fusion and segmentation processes, more accurate and faster solutions are required. Intestinal Tumours are particularly challenging for both manual and AI-based Tumour segmentations due to their intricate anatomical shapes, close adjacency to organs with similar appearances, locally variable presentations, the presence of acute non-malignant uptakes, particularly notable in patients with diabetes, and the small sizes of the Tumours.

Taking into consideration here we proposed a novel Segformer-Mix Vision Transformer segmentation algorithm for effective segmentation using of GIST from the fused CT-PET images from DIFNet. DIFNet (Deep Image Fusion Network) is a kind of CNN (Convolutional Neural Network)-based architecture that are designed to generate high-quality fused images for various combination of image modalities. The resultant fused image has very low Edge-preserving loss to retain sharp boundaries from CT and very high SSIM (Structural Similarity Index) helps in improving the boundary delineation which is useful for precise segmentation.

In the next stage for to segment the tumour region from the resultant fused from DIFNet, here we make use of Segformer a semantic segmentation model combining Mix Vision Transformer (MiT) as backbone and a lightweight MLP decoder that aggregates multi-scale features. This Segformer-Mix Vision Transformer works well with both small and large datasets and doesn’t require heavy pre-training for segmentation task with improved mean IoU, Pixel Accuracy and Dc.

**The unique contribution of the proposed model:**

1. The proposed model results effective segmentation of tumour region with improved performance metrics.
2. With the usage of DIF net based fusion the model increases delineation, there by enhances the interpretation.
3. MiT hierarchical encoder are capable of captures rich multi-scale representations without positional encodings, there by local (shallow) and global (deep) attention representations enables correct object boundary delineation.
4. The model outperforms the previous MViT architecture combinations with a high DC of 93.11, mean IoU of 0.8837, and accuracy of 97.75 pixels.

Rest of the paper is organized as follows; section 2 describes the previous works done on fusion of two image modality and segmentation of gist and their shortcomings. Section 3 illustrates the proposed works involving, the fusion of CT and PET images and Segformer-Mix Vision Transformer based segmentation. Section 4 deliberates the experimental results and conclusion in Section 5.

## 2. Literature survey

At present, many studies have been focused on segmentation of GISTs, here we present some the earlier works carried out in segmentations of tumours with various segmentation methods. Here we also present works that focus of fusion based segmentation for various tumours that are related to proposed model.

Priya R. Mohana and P. Venkatesan demonstrated an effective method for segmenting and classifying lung lesions from pet and CT image fusions using an SVM classifier that incorporates DTWT. In the first stage, lung images of both CT and PET have been deconstructed using Dual Tree m-band Wavelet Transform (DTWT) are fused using a deep learning technique. Following lung lesion segmentation, the clustering-based thresholding method is used. The last step involves extracting the texture-based and intensity-level features, which are then classified using hybrid classifiers such as Support Vector Machine (SVM), which have a 99% classification accuracy [13].

Wang, Qiong, et al. [14] proposed an algorithm to segment GISTs with an improved 3‐D U‐Net method by introducing Skip connections between encoders and decoders at different layers to account for the obvious differences in tumour size between different cases. This proposed model solves the problem of tradition U‐Net model, i.e., too weak to simultaneously extract the features of different scales. Here the model of small intestine segmentation is transferred to the model of GIST segmentation to overcome the difficulty of tumour labelling and the correlation between small intestine segmentation and GIST segmentation. Experiments findings show that the proposed method achieves better performance than that of the traditional U‐Net with Dice coefficient: FPR 0.7406, Dice-global:0.8407 and FPR:0.1786.

In 2017 Uzelaltinbulat, Selin, and Buse Ugur, proposed a lung tumour segmentation algorithm from the CT image based on medical image processing. Here the processed CT image is segmented by applying the thresholding method. In the initial stage Image pre-processing is carried out with some enhancement techniques to enhance the image quality and reduce noise in images helps for accurate segmentation. To segmenting the tumour, various regions of the images are separated in the following step. The threshold method is then used to segment the Tumour region of all images based on the gray-level intensities in each image. The model results accurate segmentation of tumour region with Accuracy of 92%, Sensitivity 91% and high Specificity 100%. [15]

For segmentation of gastrointestinal stromal Tumour segmentation from endoscopic ultrasound (EUS) with Multi-task refined boundary-supervision U-Net (MRBSU-Net) was proposed by Li, Xinyi, et al in 2020. To over the difficulty of traditional U-Net model in accurate segmenting the gists from EUS images that has diverse size, heavy shadow and ambiguous boundary. In this work we make use of multi-task re ned U-net (RU-net) to deal with diverse size and heavy shadows of gists and to solve the ambiguous problem refined boundary-supervision U-net (RBSU-net) is also designed. This RBSU-net focuses on segmenting the region on the up-sampling path and identifying the boundary in the down-sampling portion. Overall, the model provides high dice similarity coefficient of 0.92, with model cost only 0.09 sec per image in testing stage [16].

Using deep learning, Iwasa, Yuhei, et al. automatically segment the pancreatic tumours from contrast-enhanced endoscopic ultrasound video images to improve the diagnostic capability. Here as ground truth, pancreatic tumours were manually segmented from B-mode images. U-Net was used to do automatic segmentation over 100 epochs, and 4-fold cross-validation was used to assess the results. The model performance was analysed with the concordance rate that was calculated using the intersection over union (IoU). The proposed model results decent concordance rate with median IoU for all cases was 0.77 only, in auto segmentation of pancreatic tumours [17].

Zhang, Yuhuan, et al. in 2025 proposed Semi-supervised Training based Mutual Consistency Dual-stream Tumour segmentation network aiming in leverage different types of data to enhance the accuracy of GIST segmentation. To improving the accuracy of model training a semi-supervised training strategy is employed firstly, later dual-stream decoder is designed to decompose semantic information into edge flow and morphology flow. This supervised approach enables the participation of unlabelled image samples in training. And the superiority model performance is evaluated using five metrics and model results Dice coefficient- 0.762, IoU- 0.667, Precision-0.902, Jaccard-0.640, and Recall-0.740 respectively [18].

Torkaman, Mahsa, et al [19]. In 2025 proposed an organ-focused 3D CNN approach to investigate the impact of training data homogeneity on the segmentation results of intestinal tumours. The performance of the model is compared with results of previously published whole body training approach. Where Both approaches were trained using diffuse large B cell (DLBCL) patients from a large multi-centre clinical trial (NCT01287741). The proposed model results Dice score of 0.78(±0.21), Precision −0.91 ± 0.04, Recall – 0.85 ± 0.05, F1 −0.88 ± 0.04 which is comparatively higher than whole-body approach.

Torkaman, Mahsa, et al [20] in 2024 proposed Gastro-intestinal lesion segmentation using deep learning.Presented an argonbased segmentation method that uses convolutional neural networks (CNNs) to examine the effect of training data homogeneity on intestinal Tumour segmentation outcomes.To analyze the model performance two most important quantitative metrics like dice score and F1 scores are utilised here. This organ-based approach outperforms in segmentation of intestinal tumours with grater dice score (mean±std) of 0.78 ± 0.21 which is comparatively higher than whole-body approach which results only (mean±std) of 0.63 ± 0.30 and organ-based approaches generated a dice score (mean±std) of 0.63 ± 0.30 for the whole-body approaches respectively with *p*-value less than 0.0001. the proposed model also results, F1 scores of 0.86 which is higher than whole-body approach of 0.79.

Using the UW-Madison GI tract dataset, which consists of 38,496 scans, Wang, Yang-Yang, et al. investigate several encoder and decoder combinations to segment the stomach, large bowel, and small bowel in MRI images. Encoders ResNet50, EfficientNetB1, MobileNetV2, ResNext50, and Timm_Gernet_S were paired with decoders UNet, FPN, PSPNet, PAN, and DeepLab V3+ to carry out segmentation tasks in this work. For the combination of ResNet50 with DeepLab V3 + the model results high Dice value of 0.9082, an IoU value of 0.8796, and a model loss of 0.117 which is comparatively better than the other combinations of encoders and decoders [21].

Jiang, Xin, et al. [22] proposed innovative transformer-based medical image segmentation architecture called BiFTransNet, which introduces a BiFusion module into the decoder stage, enabling effective global and local feature fusion by enabling feature integration from various modules. Furthermore, a multilayer loss (ML) technique is used to track the learning process of each decoder. The suggested approach obtained an intersection-over-union (IoU) score of 86.54% and a dice score of 89.51% on the UW-Madison Gastrointestinal Segmentation dataset. Compared with the state-of-the-art methods, the proposed method achieves superior segmentation performance in gastrointestinal segmentation tasks.

In 2025, Sharma, Neha, et al. used the UW-Madison dataset to develop a hybrid model for different encoder–decoder variants for semantic segmentation of the gastrointestinal tract. The proposed make use of ResNet50, EfficientNetB1, MobileNetV2, ResNext50, as encoders in combination of decoders like UNet, FPN, PSPNet, PAN, and Deep Lab V3+ for segmentation of gi tumour. Finally, it was demonstrated that, in comparison to all other combinations, the combination of Deep Lab V3+ and ResNet 50 successfully segmented the tumour region of an MRI image with a dice value of 0.9082, an IoU value of 0.8796, and a model loss of 0.117 [23].

In 2026 Huang, Chenshen, et al. suggested a novel graph-based framework called Geometric Multi-Instance Learning (Geo-MIL), which explicitly models the spatial interactions between tissue patches. The segmentation job, which is the main emphasis of this paper, demonstrates the greatest benefit of Geo-MIL with Dice score of 0.789 [24].

Using a publicly accessible data set, Gupta, Pankaj, et al. published a Pancreatic Tumours Segmentation Model employing Endoscopic Ultrasound in 2026 to assess the effectiveness of a Deep Learning model. Here is a Vision Transformer-based deep learning segmentation model for tumours of the pancreas. Additionally, the model included preprocessing techniques including

cropping, resizing to 512x512 pixels, and grayscale conversion. With DSC of 0.657, IoU of 0.614, sensitivity of 71.8%, and specificity of 97.7%, the proposed approach showed excellent performance for pancreatic tumour segmentation in EUS images [25].

Pathak, Disha Mohini, et al. In 2026 proposed medical image fusion using deep learning with transformers with Optimal feature selection. The proposed hybrid fusion method combines Convolutional Neural Network (CNN) with Swin Transformer (ST) architectures for to improve feature representation one of the major limitations in image fusion. Furthermore, a novel PCA-based block is demonstrated to choose high-gradient features, improving image quality and producing superior fusion outcomes with a little feature component. With the lowest mean metric error score of 9.99, the proposed approach performs better compared to all other models. [26]

In 2024 for medical image fusion an ensemble deep learning model using Siamese neural networks and VGG-19 was proposed by Allapakam, Venu, and Yepuganti Karuna [27]. The proposed ensemble model with the use of SNN in combination with VGG-19 leverages the advantage of both the architecture, making the model more powerful in the effective fusion of various source images. SNN has two identical networks which consist of convolution and 2hidden layers help to extract hierarchical features. On the other hand, the Vgg-19 model is convolutional neural, which is already pre-trained by millions of images from ImageNet. For to produce a fused image quality with improved visual quality and performance metrics are attained by make use of these two powerful networks which are familiar for feature extraction [27].

From the above works few research gaps were observed like, the improved 3‐D U‐Net method results very low Dice coefficient: FPR 0.7406, Dice-global:0.8407 FPR:0.1786 [14]. Threshold based segmentation results very low Accuracy of 92 and Sensitivity of 91 [15]. The U-Net variations introduced in [18] are not universally applicable in a variety of medical imaging tasks, especially in more challenging and noisy data. Segmenting complex, irregular tumours especially GISTs with higher accuracy is always challenging task

## 3. proposed model

To overcome above limitation and to improve the segmentation accuracy, here we proposed a two-stage segmentation approach. In the initial stage to improve the image interpretation an DIF-Net based image segmentation is proposed to fuse CT and PET images of GISTs. Later for effective segmentation an Segformer-Mix Vision Transformer that combines both CNN and Transformer, enabling better understanding of the full image and fine details—ideal for segmenting complex, irregular tumours like GISTs. It is computationally efficient and requires fewer parameters than many hybrid or transformer-only models, making it more practical for real-world clinical use, especially with limited GPU memory. Segformer doesn’t rely on position embeddings, which makes it more robust to variations in tumour location and shape, important in real clinical imaging where GISTs may appear in diverse regions. The suggested model produces high-quality fused images with better interpretation and is more successful at accurately segmenting tumour regions of different sizes and shapes.

The proposed approach for the fusion and segmentation of CT-PET images using DIF-Net and MViT encoder-based segmentation is discussed in this session. Here we present the utilization of DIF-Net, which is a deep learning framework for combination of CT and PET images where both modalities convey complementary information. DIF-Net employs advanced fusion techniques that preserve the anatomical and functional details of the fused image with improved image quality.

**Fig 1:**
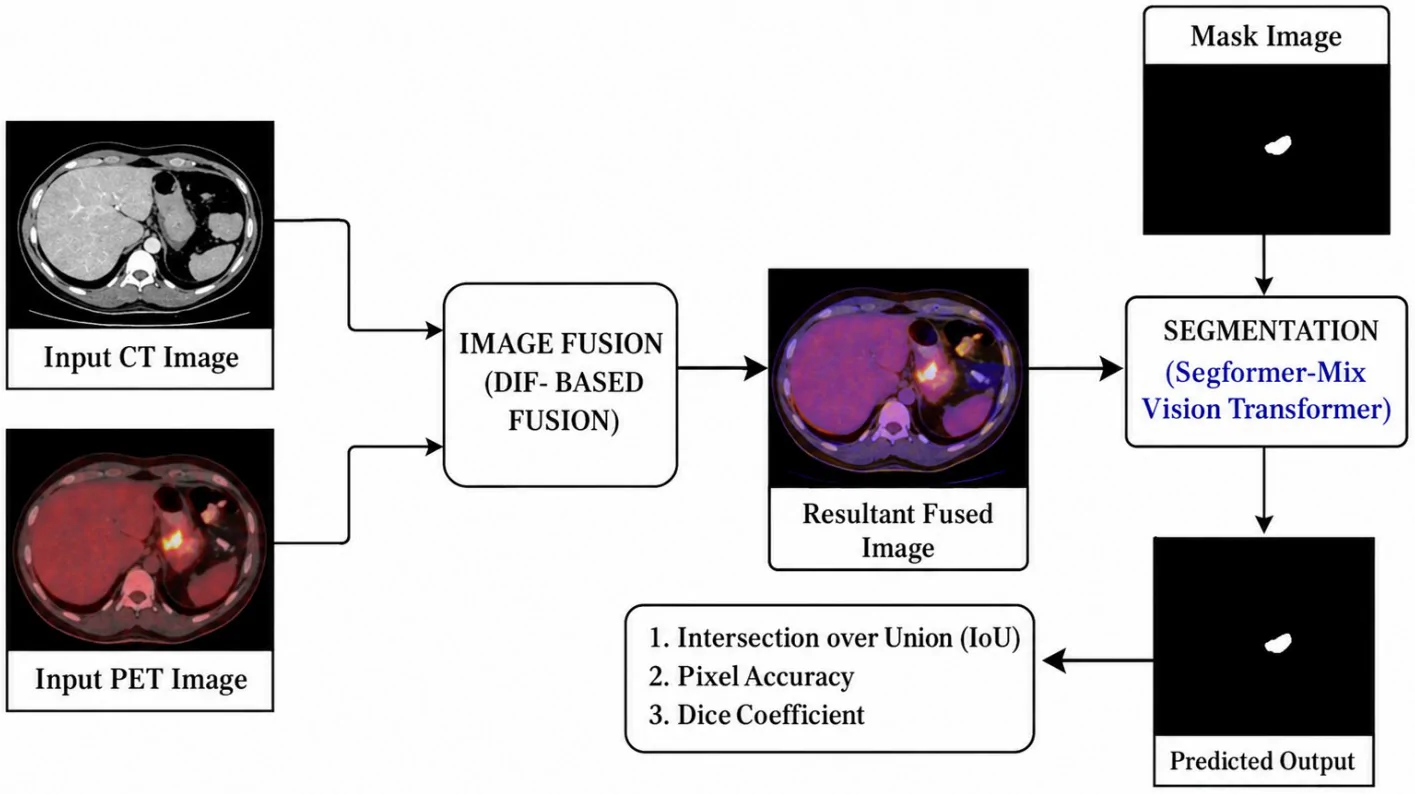
Proposed Hybrid Framework for GIST Tumour Segmentation from CT and PET Images.

In the next stage, Segformer architecture with MViT encoder is used for pixel-level segmentation. It uses a transformer-based encoder, enabling it to efficiently extract local and global features from the fused CT-PET data, allowing for efficient segmentation of critical structures such as tumours and lesions. This consistency between fused and segmented form helps the model to yield a better, robust and accurate tumour segmentation for improved diagnosis. Fig 1 illustrates the proposed model architecture.

This work’s primary innovation is the unified framework for GIST analysis that combines SegFormer-based transformer segmentation and multimodal medical picture fusion. The proposed technique makes use of fused multimodal data to improve the effectiveness of transformer-based feature learning, whereas existing research mostly concentrates on either image fusion or transformer-based segmentation separately. This integration preserves computational efficiency while enabling better segmentation performance, more precise tumour border location, and enhanced contextual representation. The proposed framework therefore provides a practical and effective solution for multimodal gist tumour segmentation.

### 3.1. Proposed DIF-net model for fusion

**Fig 2:**
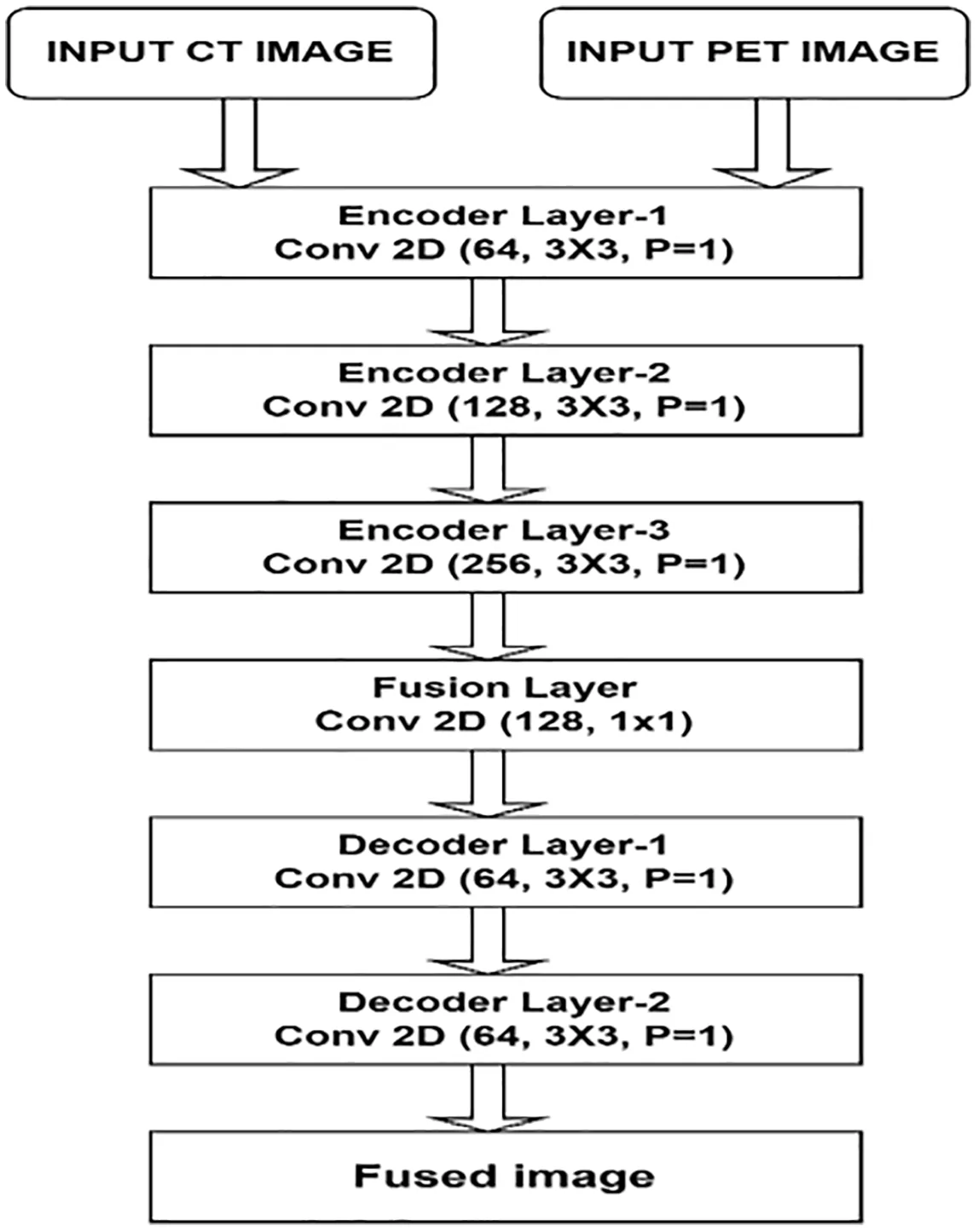
Fusion of CT-PET Images with DIF-Net Architecture.

Here we proposed a deep learning model “DIF-Net: Deep Image Fusion Network” for fusing CT and PET medical images into a fused image. DIF-Net uses convolutional neural network (CNN) layers that learn to effectively integrate the complementary anatomical and functional content in CT and PET images. The proposed DIF-Net network consists of dual encoders, cross-attention, fusion block and decoder paths to store important spatial and structural information can be seen from Fig 2. In DIF-Net, we employ ReLU activation functions following each convolutional layer in both the encoder and decoder networks to improve nonlinear feature representation. Here, the Adam optimizer is used to train the model with an initial learning rate of 0.001, ensuring stable convergence and effective parameter optimization. Effective feature extraction, feature fusion, and picture reconstruction throughout the fusion process are facilitated by these design decisions.

This fusion network offers fused image with balanced anatomical information (CT) and metabolic contrast (PET) and Cross-modality attention preserves. In this work the CT and PET images of GISTs are obtained from publicly available data sets from GitHub - Praneet Mohan/GIST-CT-PET: GIST CT PET images · GitHub. Here we consider both CT and PET images of various gastrointestinal stromal tumors.

The proposed model also offers fused image with High-resolution detail with improved image quality helps in exact tumour localization, improved diagnostic interpretation and automatic tumour segmentation.

#### 3.1.1. Encoder layers.

- **Encoder Layer 1:** A conv layer, 64 filters, kernel size 3, padding 1. This unit captures low-level features from the fused CT and PET images.
- **Encoder Layer 2:** Like the first encoder layer, adding a convolutional layer with 128 filters, kernel size 3, and padding 1 to further extract features.
- **Encoder Layer 3:** A conv layer of 256 filters, kernel size 3, padding 1, captures high-level and more abstract features, which is suitable for integration.

#### 3.1.2. Fusion layer.

- It employs a convolution layer with 128 filters and kernel size 1 to merge the encoded features That takes modality-specific characteristics of CT and PET as input and generates fused features.

#### 3.1.3. Decoder layers.

- **Decoder Layer 1:** A conv layer, 64 filters, kernel size 3, padding 1. It starts reconstructing the spatial information of the fused image.
- **Decoder Layer 2:** Consists of a convolutional layer with 1 filter, padding = 1 and kernel size = 3 which will finally convert the reconstructed fused image into a 1-channel output.

Given two input images, $I_{CT}\;$and $I_{PET}$, the fusion process within DIF-Net can be mathematically represented as follows:


$$\begin{array}{c} \;F_{CT}=\;E_{CT}\left(\;I_{CT}\right) \end{array}$$ (1)

$$\begin{array}{c} F_{PET}=\;E_{PET}\left(\;I_{PET}\right) \end{array}$$ (2)

Where $E_{CT}\;$and $E_{PET}$ are encoding functions consisting of convolutional layers designed to extract modality-specific features from CT and PET images respectively. Next, these encoded features are fused using a feature-level fusion operation, typically implemented via concatenation followed by convolution:


$$\begin{array}{c} F_{fused}=Conv(Concat\left(F_{CT},F_{PET}\right) \end{array}$$ (3)

Here, Concat(.) concatenates the feature maps along the channel dimension, and Conv(.) is a convolutional layer that integrates and refines the combined features. The fused features $f_{fused}$ are then passed through a decoding path to reconstruct the final fused image $I_{fused}$:


$$\begin{array}{c} I_{fused}=D\left(F_{fused}\right) \end{array}$$ (4)

**Step 1:** Input CT and PET images:

- Input $I_{CT}$,$I_{PET}$

**Step 2:** Encode input images independently:

- Compute $F_{CT}=\;E_{CT}\left(I_{CT}\right)$
- Compute $F_{PET}=\;E_{PET}\left(I_{PET}\right)$

**Step 3:** Perform feature-level fusion:

1. Compute $F_{fused}=Conv(Concat\left(F_{CT},F_{PET}\right)$

**Step 4:** Decode the fused features to reconstruct the fused image:

2. Compute $I_{fused}=D\left(F_{fused}\right)$

**Step 5:** Output the fused image:

3. Compute $I_{fused}$

Where D(.) represents the decoding function composed of transposed convolutional layers, reconstructing the spatial dimensions and synthesizing the final fused image. The algorithm steps of DIF-Net are as follows:

### 3.2. Proposed Segformer with MViT Encoder

**Fig 3:**
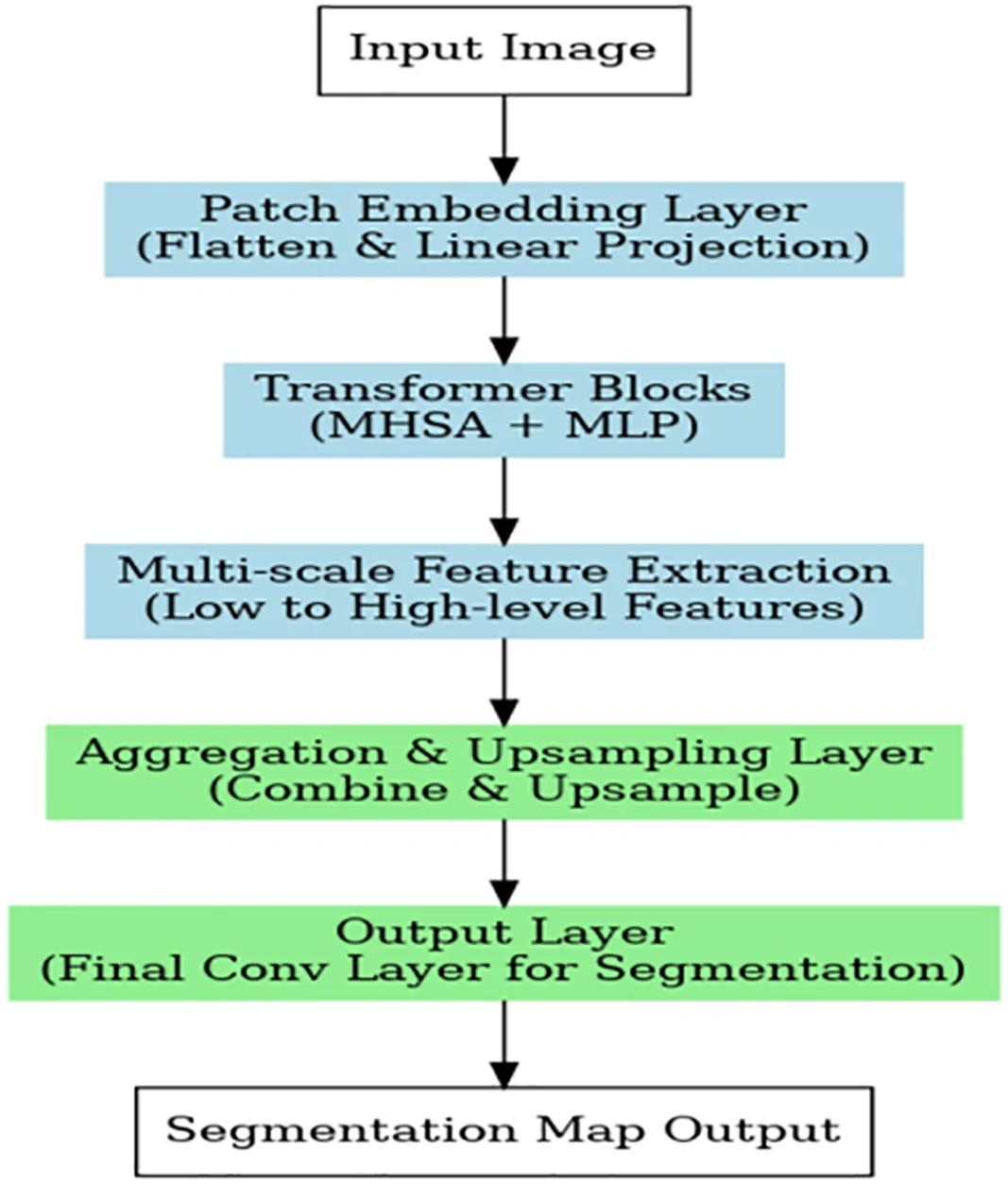
Layers in Proposed Segformer Architecture.

Segformer is great at capturing local texture, spatial patterns and excel at modelling global context and long-range dependencies. The Segformer, enhanced with the Mix Vision Transformer (MViT) encoder, provides advanced capabilities for accurately segmenting fused CT-PET images. MViT encoder efficiently captures both local and global contextual information, significantly improving segmentation performance. The proposed Segformer architecture comprises several layers which depicted in Fig 3.

#### 3.2.1. Mix vision transformer encoder layers.

- **Patch Embedding Layer:** The input image is divided into smaller non-overlapping images. The image is broken down into patches, and each patch is flattened into a one-dimensional vector. A linear projection of these vectors yields an embedded token representation, allowing the model to manage spatial configurations as routine inner representations, amenable to transformer architectures.
- **Transformer Blocks:** Stacking multiple transformer layers, where a single transformer layer is multi-head self-attention (MHSA) followed by multi-layer perceptron’s (MLP), to learn the global context and interactions between different image patches.
  - **Multi-Head Self-Attention (MHSA):** This module allows the network to weigh the importance of different patches relative to each other, effectively capturing long-range dependencies and global contextual relationships within the image.
  - **Multi-Layer Perceptron (MLP):** Following MHSA, an MLP module refines the embeddings, capturing complex, nonlinear transformations of the features to enhance the discriminative ability of the representation.
  - **Multi-scale Feature Extraction:** SegFormer has a unique approach to multi-scale feature extraction. The transformer blocks work at many resolutions or scales. Stages at lower levels are responsible for extracting fine-grained features, such as edges and textures and detailed boundaries, and stages at higher levels capture more abstract, global context information that is more relevant for differentiating semantic categories with a broader scope. Take -- Multi-Scale Feature Integration: Integrating features across different spatial scales to ensure robust understanding and representation of diverse information.

#### 3.2.2. Decoder Layers.

- **Aggregation and Up sampling Layer:** It merges features extracted from different stages of the transformer. This layer aggregates the information at different scales to retain fine detailed low-level visual information as well as the decoded high-level visual context range. It then up-samples these spatially aggregated features to return them to the original input image resolution, ensuring accurate localization.
- **Output Layer:** The last convolutional layer that takes the upscaled features and computes a fully connected segmentation map. Here each pixel of the map is labeled as different semantic parts or classes defining segmented spaces. The segmentation map produced organically maintains the dimensional integrity of the original photo, the borders are sharp, and the segments are informative, matching the semantic classes.

Let $I_{fused}\;$denote the fused input image. The segmentation process using Segformer with MViT encoder can be expressed as:

First, the input image undergoes multi-scale encoding:


$$\begin{array}{c} F_{enc}={MViT}_{encoder}\left(I_{fused}\right) \end{array}$$ (5)

The multi-scale encoded features $F_{enc}\;$are then decoded to produce the segmentation map:


$$\begin{array}{c} {S=Decoder\;(F}_{enc}) \end{array}$$ (6)

Where S is the final segmentation output, delineating regions of interest accurately.

**Step 1:** Input fused image from DIF-Net:

[1] Input $I_{fused}$

**Step 2:** Encode the fused image using MViT encoder:

• Compute $F_{enc}={MViT}_{encoder}\left(I_{fused}\right)$

**Step 3:** Decode the encoded multi-scale features to produce segmentation:

1. Compute ${S=Decoder\;(F}_{enc})$

**Step 4:** Output the segmentation map:

2. OutputS

## 4. Simulation results and discussion

### 4.1. Data set

This section presents the experimental results carried out to validate the proposed model. CT and PET images are first fused, and then semantic segmentation is performed on the fused image. These experiments were carried out on publicly available data set from GIST-CT-PET/DB.zip at main Praneet Mohan/GIST-CT-PET · GitHub, that consists of 60 CT and PET images. The images are of size 256 x 256 that are subjected with various image preprocessing techniques like rotation, flip, crop and scale which Increases the dataset to 200 images in terms both quality and quantity to enhance model robustness and mitigate overfitting. These 200 images were split into training 140 images, validation 30 images and testing 30 images subsets corresponding to 70%, 15% and 15% of the data respectively.

### 4.2. Experimental setup

The experiments are conducted in Google Colab Platform—Python 3 Google Compute Engine backend (GPU). Training was performed with a batch size of 16 for 10 epochs using the Adam optimizer with a learning rate of 0.001. The allotted system RAM is 12 GB; GPU RAM is 15 GB, and hard disk size is 166 GB. The average training time was approximately 4–5 hours. These settings were selected to ensure stable convergence and optimal segmentation performance.

**Fig 4:**
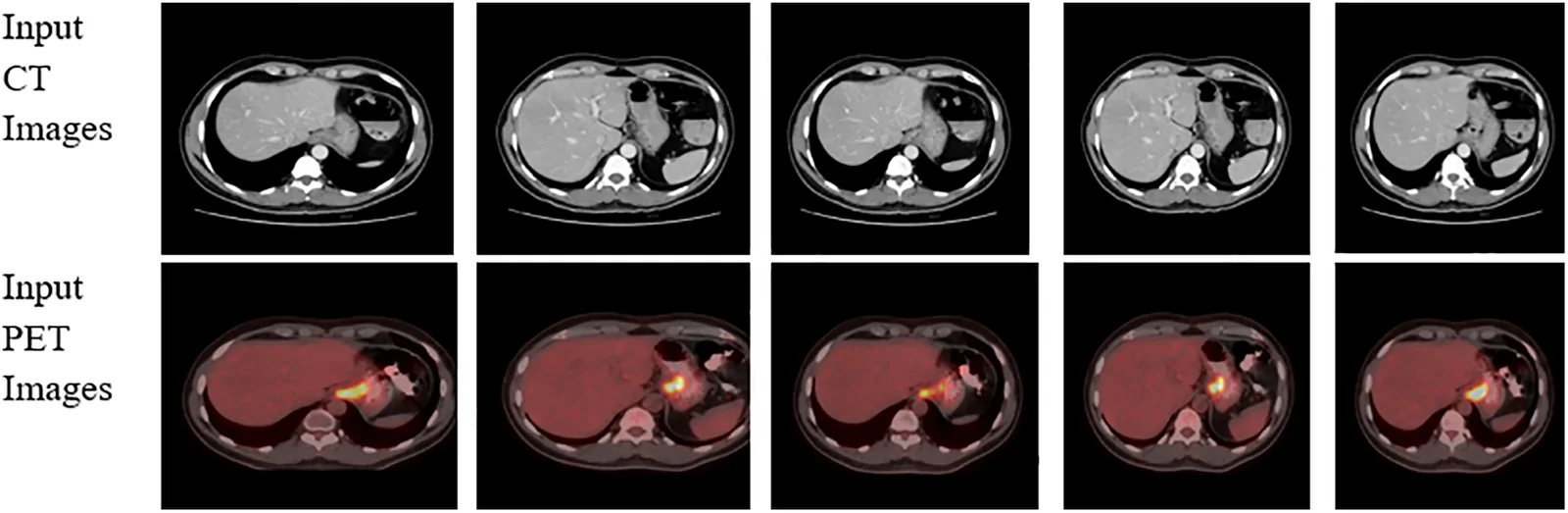
Sample gist CT-PET images from data set.

The model was trained on a combination of CT and PET images, with the following experimental settings:

- **Input Images**: The input provided was 256x 256 pixels, CT and PET of 200 pairs as shown in Fig 4 sample images.
- **Model Architecture**: The Segformer with an MViT encoder was used for segmentation after the DIF-Net model included both CT and PET characteristics using a 2-channel input. Labels for several areas of interest (ROIs), including tumours, were generated by the segmentation result.
- **Loss Function**: We used the L1 loss (Mean Absolute Error) to compare the fused image with the simple averaging fusion approach.
- **Optimizer**: Model was optimized using Adam optimizer which had learning rate of 1e-3.

### 4.3. DIF-net model training analysis

**Fig 5:**
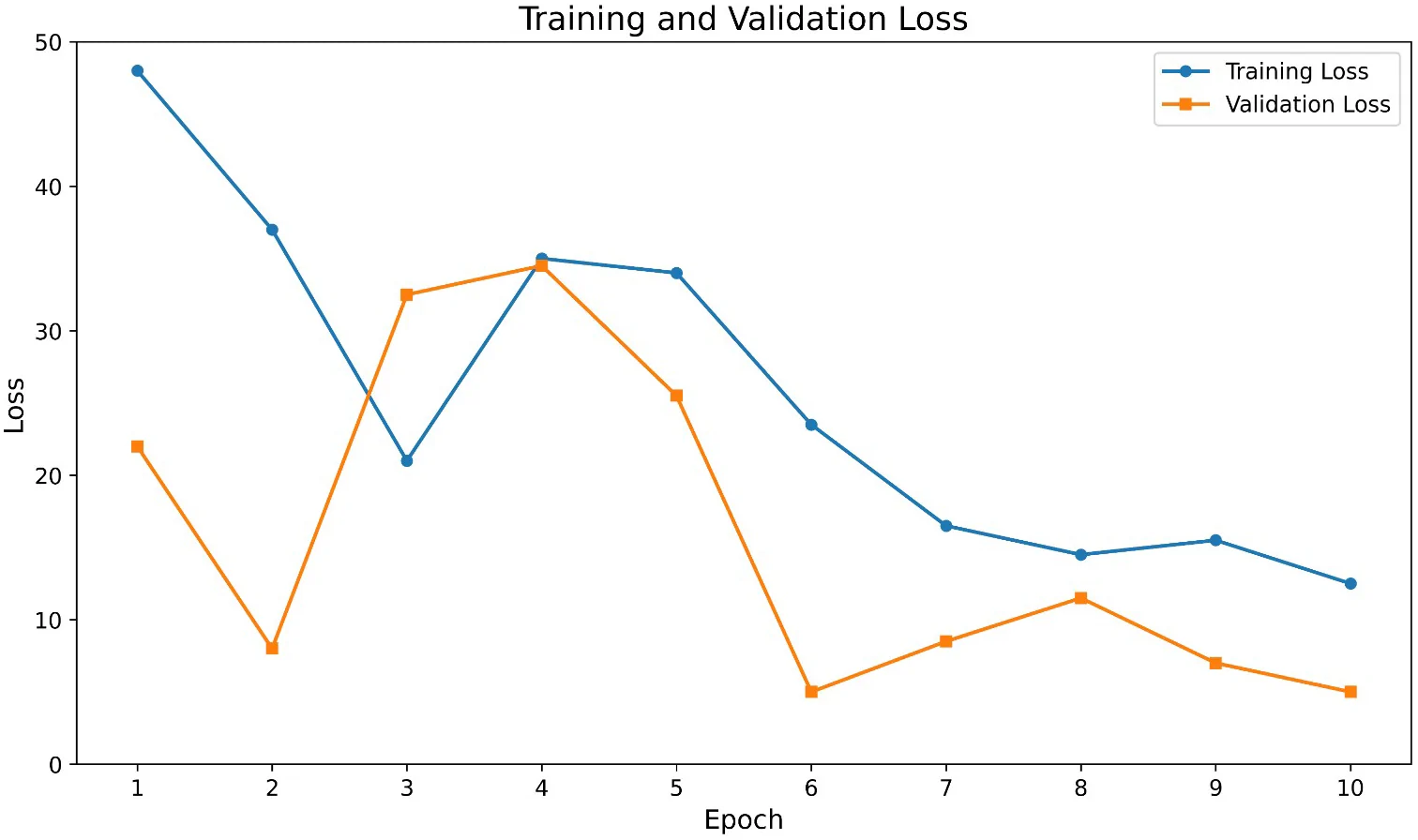
Training and validation loss plot of the proposed DIF-Net model.

The proposed DIF-Net fusion model trained to combine CT and PET image for better segmentation. The dataset is split into 70% training and 30% testing + validation. This model uses the high-resolution anatomical details provided with CT scans, but it also includes functional information provided with PET scans, to produce more accurate tumour segmentation. To measure the model’s performance, we monitored the training and validation losses 10 epochs. In this Example there is a plot that shows how the training got fancy, How the model can learn and make sense of the real images to generalize about the new unseen image which makes it very efficient and robust. The proposed DIF-Net model training and validation loss plot is as shown in Fig 5.

At first, training loss is high at about 50, which is usual for the early experiences of training a model. This loss is high because the model does not fit the data very well at this stage. But the loss decreases over the time of training, which is something you expect for a machine learning task. Except for epoch 0, the drop in training loss is quite uniform until epoch 4, and then the rate of drop reduces. This indicates that the model is approaching convergence. This phenomenon demonstrates the model’s ability to lessen its mistake over time as well as its capacity to learn from its data.

The validation loss (orange line) starts higher compared to training loss and until now follows a similar decreasing pattern as training loss. But look at the validation loss, it is more fluctuated, which is expected because the validation data has a more variability, which was not seen by the model during the training phase. From epochs 5 and 6 onward, the validation loss stabilizes and begins to decrease more consistently after epoch 7. A consistent downward trend shows that model is generalizing well over unseen data which is a desired behaviour for real-life environments.

The training loss, as is the case in typical training, stays lower than the validation loss all through training. The lower validation loss indicates that the model is overfitting the training data and not generalizing well, while the slightly higher validation loss indicates that the model has adapted to the more complex diverse validation set. But both the losses keep steadily decreasing, while their proximity to each other shows us that the model is not overfitting, which is good! Overfitting can be seen when the training loss decreases, but the validation loss increases, and this is not the case here.

Finally, both the training loss and the validation loss have stabilized to low values around epoch 10 indicating convergence in the model. Which means the model learnt a best model to fuse CT and PET images keeping in mind training accuracy and generalization performance. The gradual reduction of losses as well as the stabilization seen late in the training is typical of a well-optimised model where good performance on both fitting well to data and generalisation to unseen data is achieved.

### 4.4. Performance evaluation metrics for image fusion

To evaluate the performance of the image fusion process, we use various Fusion Evaluation Metrics that assess the quality of the fused image in terms of both structural similarity and information content. Here’s an explanation of each metric and how they can be used to evaluate the performance of the proposed DIF-Net model for CT-PET image fusion:

1. **Peak Signal-to-Noise Ratio (PSNR)**: it is used to analyse the quality of the fused image by comparing it with the original image. A larger PSNR means an improved quality because there are less differences between the fused image and the input images.

$$\begin{array}{c} PSNR=\text{1}\text{0}\times {log}_{\text{1}\text{0}}\left(\frac{{MAX}_{I}^{\text{2}}}{MSE}\right) \end{array}$$

Where ${MAX}_{I}$ is the maximum possible pixel value of the image, and MSEis the Mean Squared Error between the original and fused image. A higher PSNR value indicates that the fused image retains more information from the original scan.

2. **Structural Similarity Index (SSIM):** Quantifies the perceptual distortion by comparing the luminance, contrast and structure of the fused image with the original input image. SSIM ranges from a value of -1 (no similarity), to 1 (identical).

$$\begin{array}{c} SSIM(x,y)=\frac{\left({\text{2}\mu }_{x}\mu_{x}+C_{\text{1}}\right)\left({\text{2}\sigma }_{xy}+C_{\text{2}}\right)}{\left(\mu_{x}^{\text{2}}+\mu_{y}^{\text{2}}+C_{\text{1}}\right)\left(\sigma_{x}^{\text{2}}+\sigma_{y}^{\text{2}}+C_{\text{2}}\right)} \end{array}$$

Where, $\mu_{x},\;\mu_{y}\;$are the means, $\sigma_{x},\;\sigma_{y}\;$are the variances, and $\sigma_{xy}$ is the covariance of the images. A higher SSIM value indicates better preservation of structural features in the fused image compared to the original input image.

3. **Mutual Information (MI):** Quantifies the degree of overlap between raw image and combined image information. Mutual information measures how much information from the input image is transferred to the fused output, meaning that the more MI the better the fusion.

$$\begin{array}{c} MI(x,y)=H(x)+H(y)-H(x,y) \end{array}$$

Where H(x) and H(y) are the entropies of the individual images, and H(x,y) is the joint entropy. A higher *MI* value indicates that the fused image retains more relevant information from the original input image.

4. **Universal Image Quality Index (UIQI):** A perceptual quality metric that evaluates an image’s quality by comparing its luminance, contrast, and structure. The second metric, UIQI is a very important feature for evaluating the perceptual quality of the fused image.

$$\begin{array}{c} UIQI=\frac{{\text{4}\mu }_{x}\mu_{y}\sigma_{xy}}{\left(\mu_{x}^{\text{2}}+\sigma_{x}^{\text{2}}\right)\left(\mu_{y}^{\text{2}}+\sigma_{y}^{\text{2}}\right)} \end{array}$$

Where $\mu_{x},\;\mu_{y}$ are the means, $\sigma_{x\;},\sigma_{y}$ are the standard deviations, and $\sigma_{xy}\;$is the covariance. A higher *UIQI* value suggests that the fused image has better perceptual quality in terms of structural similarity and noise reduction.

5. **Edge Preservation:** It estimates the quality of the fused image, retaining its edges of the original input image. This is important for maintaining anatomical structures in medical imaging. This can be measured through the edge-detection algorithms (Sobel or Canny operator) and edge maps of the fused and original images can be compared. The higher the edge preservation score, the more important edges are preserved in the fused image, helping the integrity of anatomical detail.

### 4.5. Results for image fusion

**Fig 6:**
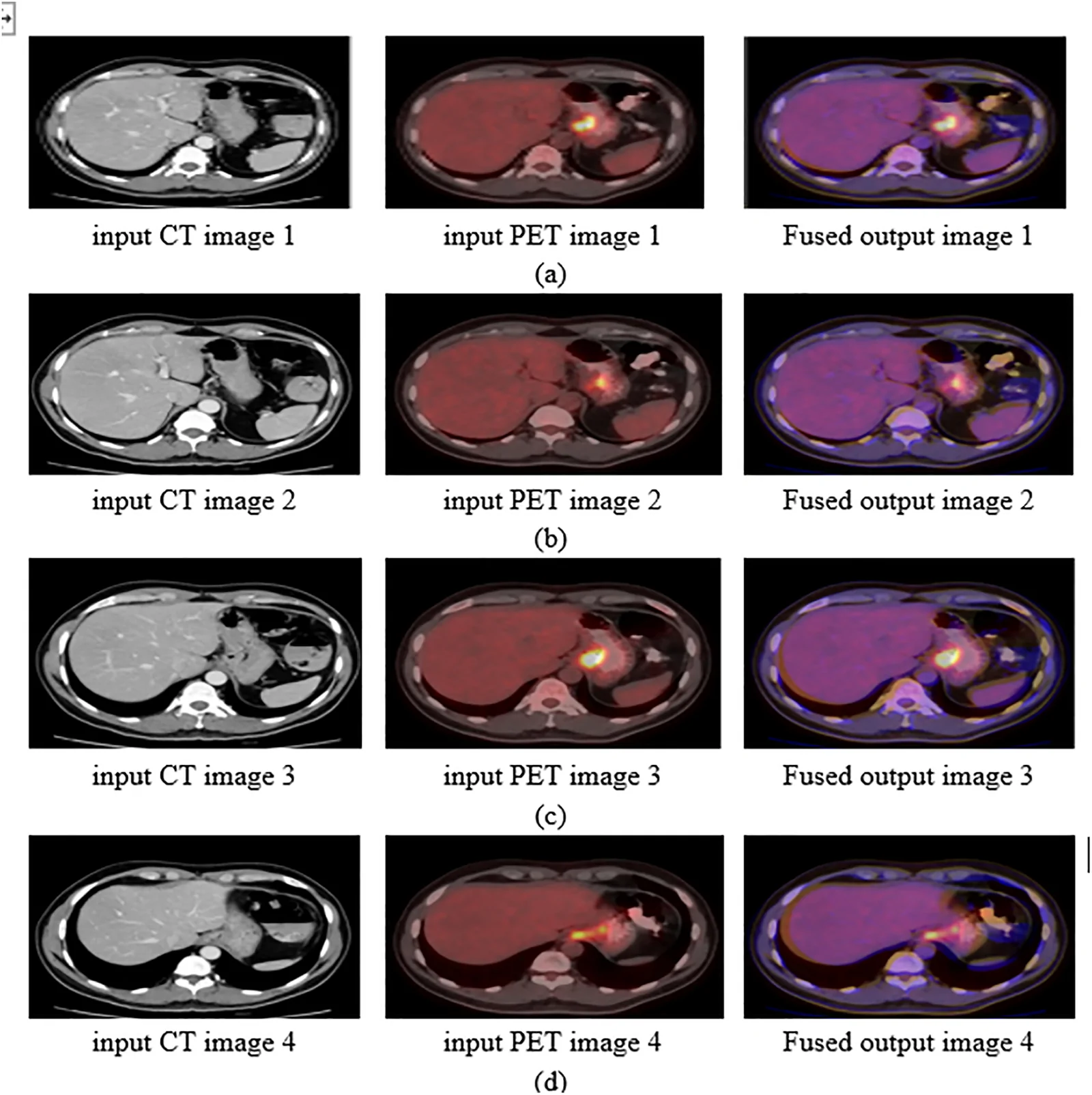
Resultant Fused images of Proposed DIF-Net model.

The Fig 6 shows the resultant fused image of CT-PET using DIF-Net. High-resolution anatomical information obtained from CT scans and exact functional details from the PET scans were retained in the fused pictures created with DIF-Net, demonstrating exceptional structural and functional detail integration.

The performance evaluation metrics on CT images for the DIF-Net fusion model are reported in Table 1. PSNR (Peak Signal-to-Noise Ratio), SSIM (Structural Similarity Index), MI (Mutual Information), UIQI (Universal Image Quality Index) and Edge Preservation are some metrics which encompasses the entire image to represent image quality, structure and the information retained along with edge preservation.

**Table 1:** Performance Evaluation Metrics w.r.t CT images.

Image	PSNR	SSIM	MI	UIQI	Edge
CT Image-1	18.6537	0.7403	6.5842	0.9037	0.8289
CT Image-2	18.5895	0.7527	6.6584	0.9028	0.8278
CT Image-3	18.6190	0.7513	6.6509	0.9027	0.8264
CT Image-4	18.9146	0.7576	6.2671	0.8986	0.8223

The PSNR values from CT images are between 18.5895 and 18.9146, showing that multispectral images are still high quality with less distortion. So, higher PSNR indicates that images are meaningful and less noise. Regarding SSIM, or structural similarity, we obtain the values from 0.7403 to 0.7576 showing that the CT images structures have integrity and closely resembling ground truth. The MI (Mutual Information) values extracted from the processed whole mount images are between 6.2671 and 6.6584, which shows that the images have lost little information in the anatomical and functional details during the processing. The UIQI (Universal Image Quality Index) scores between 0.8986 and 0.9037 prove that CT images retain their strong perceptual quality about the luminance, contrast, and structure. Lastly, the Edge Preservation, with a range of 0.8223 to 0.8289, confirms that the images successfully maintain important anatomical edges, a crucial factor for accurate medical assessment and diagnosis.

The PET image performance evaluation index details of the DIF-Net fusion model are depicted in Table 2. These metrics are PSNR (Peak Signal-to-Noise Ratio), SSIM (Structural Similarity Index), MI (Mutual Information), UIQI (Universal Image Quality Index) and Edge Preservation. The values of PSNR of the PET images 18.2216–18.6147, as well reflects the overall image quality in terms of noise and preservation of important details in images. These values suggest PET images had an acceptable quality with less distortion in the whole fusion process. Outcomes of SSIM, a metric that gauges structural similarity, range from 0.7065 to 0.7404, suggesting that the PET pictures’ structural integrity is maintained albeit somewhat variable. The SSIM scores show a good degree of resemblance to the original PET pictures, although being lower than those for the CT scans. With a range of 6.7214 to 6.8791, the MI (Mutual Information) measure indicates that the PET images retain a significant amount of information during fusion; higher values signify the preservation of more functional data from the PET scans. The UIQI (Universal Image Quality Index) values vary from 0.8024 to 0.8180, where the perceptual quality of the PET images in terms of luminance, contrast and structure is represented. Although these values are lower than those for the CT images, they still reflect a good perceptual quality.

**Table 2:** Performance Evaluation Metrics w.r.t PET images.

Image	PSNR	SSIM	MI	UIQI	Edge
PET Image-1	18.3974	0.7384	6.8042	0.8180	0.4527
PET Image-2	18.2216	0.7404	6.7603	0.8086	0.4448
PET Image-3	18.2773	0.7304	6.8791	0.8151	0.4653
PET Image-4	18.6147	0.7065	6.7214	0.8024	0.4800

Table 3 shows the entropy of the fused images generated by the DIF-Net model. Entropy is a measure of an image’s information content. More information and lesser redundancy in the fused image can be indicated by higher entropy values, which is an important requirement as it reduces loss of significant features from PET and CT modalities in the fused image. The entropy values for the fused images are from 6.6925 to 6.8136, which means that the high information preserved in the fused images. The entropy value of Fused Image-3 is the most 6.8136, indicating that this fusion has gained a higher level of integrated information than other images. On the other hand, Fused Image-4 has the maximum entropy value of 6.6925, which represents that a considerable amount of information from both input images is being retained.

**Table 3:** Performance Evaluation Metrics w.r.t Fused images.

Image	Entropy
Fused Image-1	6.8036
Fused Image-2	6.7846
Fused Image-3	6.8136
Fused Image-4	6.6925

### 4.6. Segmentation results

The evaluation of the proposed model segmentation output (DIF-Net fusion, Segformer with the MViT encoder) was performed, comparing the predicted Tumour regions with the manual ground truth segmentation masks.

#### 4.6.1. Performance evaluation metrics for segmentation.

The test metrics for validation of the segmentation model are mIoU (Mean Intersection over Union) and Test Set Pixel Accuracy. These metrics offer detail into the capacity of the model correctly classify pixels and precisely define regions of interest in the images segmented.

1. ***Mean Intersection over Union (mIoU)***: As a frequent metric for semantic segmentation tasks, mIoU measures the accuracy of the predicted segmentation. Also, code uses the ground truth mask to calculate how much overlapping exists between predicted and true segmentation mask. The formula for mIoU is given by.

$$\begin{array}{c} mIoU=\frac{\sum_{i=\text{1}}^{N}Intersection\left(A_{i},\;B_{i}\right)}{\sum_{i=\text{1}}^{N}Union\left(A_{i},\;B_{i}\right)} \end{array}$$

Where $A_{i},\;B_{i}$ are the predicted and ground truth segmentation masks, respectively, for class i. *N* is the total number of classes. The intersection is the area where the predicted and ground truth masks overlap, and the union is the total area covered by both masks. Higher *mIoU* values indicate better segmentation performance, with values closer to 1.0 showing perfect overlap between the predicted and ground truth masks.

2. ***Dice coefficient:*** Dice coefficient, is a statistical measure used to gauge the similarity between two sets, ranging from 0 (no overlap) to 1 (perfect match).

The Dice coefficient is defined mathematically as follows:


$$\begin{array}{c} \text{Dice Coefficient}=\frac{\text{2}|X\cap Y|}{|X|+|Y|} \end{array}$$

3. ***Accuracy****:* The Segformer with MViT encoder achieved segmentation accuracy of 94%, suggesting the ability of the model to correctly identify the Tumour regions in most cases (Pixel accuracy indicates the proportion of correctly predicted pixels over the entire test set.) It is a straightforward measure of how many pixels the model predicts correctly regardless of class. The formula for test set pixel accuracy is.

$$\begin{array}{c} Pixel\;Accuracy=\frac{Number\;of\;Correctly\;Classified\;Pixels}{Total\;Number\;of\;Pixels\;in\;the\;Image} \end{array}$$

#### 4.6.2. Simulated results for training and testing.

**Fig 7:**
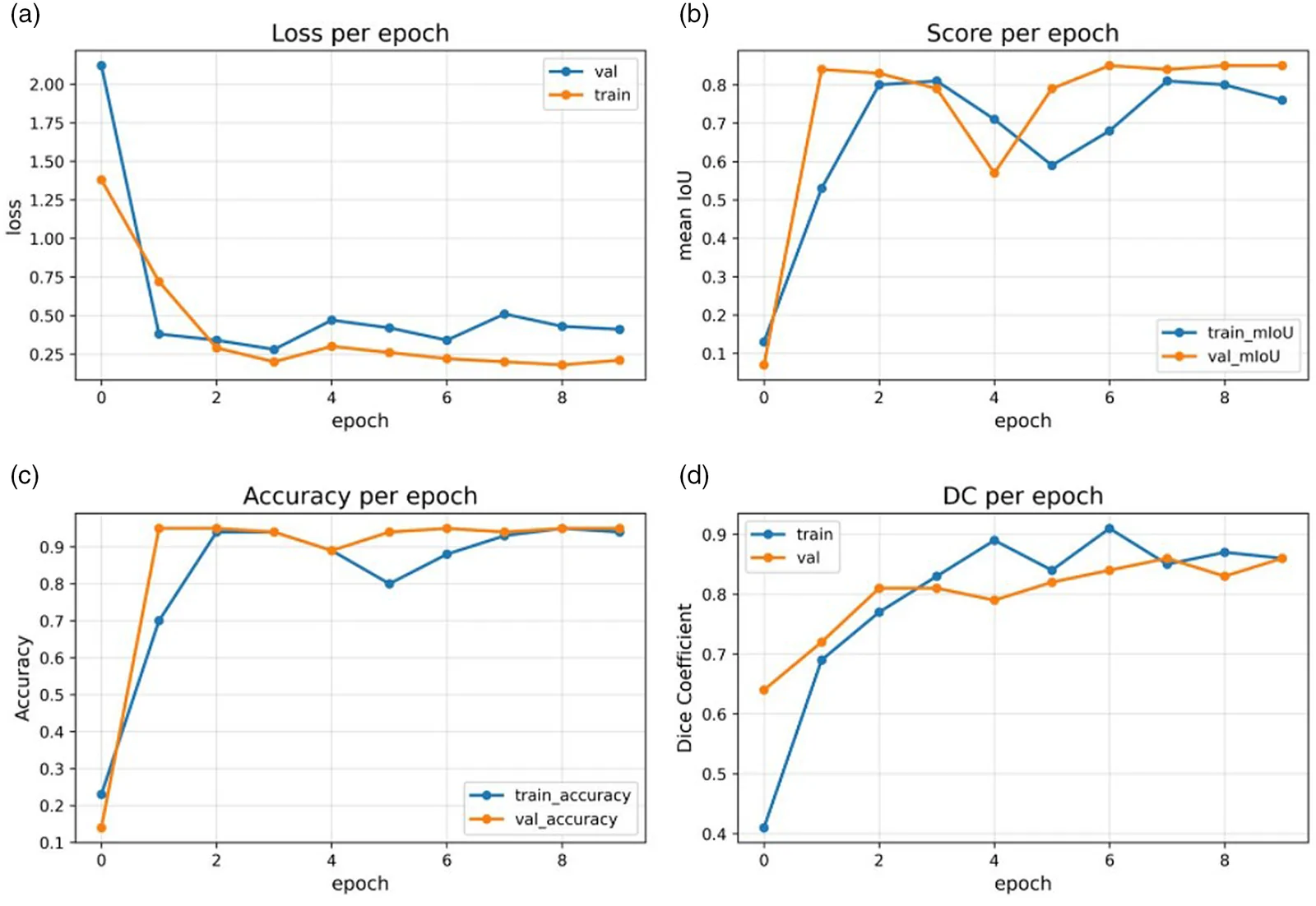
(a) Loss (b) mean Intersection Over Union (c) Accuracy (d) Dice Coefficient, Score per Epoch of segmented output.

Fig 7(a) showing Loss per epoch for the proposed segmentation model, that training loss and validation loss against 10 epochs. In the beginning, both the training and validation loss are high, with the training loss at about 1.5 and the validation loss at 2.0. As we train the model, we see that both losses fall quickly, with the training loss falling below 0.5 by the end of epoch 3, while the validation loss follows a similar trajectory. Starting from the later epochs (epochs 6–8) both the training and validation losses go around the same value which means the convergence of the model. The validation loss is always marginally higher than the training loss, which is an expected observation because it indicates that our model fits to the training data without overfitting the data (it’s generalizing successively to the validation data). Plot shows its working properly, improving steadily on the training set as well as on the validation set, error consistently shrinking across epochs.

Fig 7(b) illustrates the average IoU (Intersection out of Union) score per epoch achieved with the suggested segmentation model. These initial conditions yield low training mIoU and validation mIoU (both begin at approximately 0.1). The training and validation mIoU improve as training progresses, eventually hitting over 0.8 by epoch 2. The validation mIoU increases, but is more erratic (e.g., between epochs 3 and 5) before it too settles. Note: The training mIoU is always larger/better than the validation mIoU, which is normal behaviour and expected due to some overfitting on the training data. In general, the plot shows nice learning without problems, as both training and validation performance improve across the epochs.

Fig 7(c) describes the accuracy for each epoch concerning the suggested procedure for segmentation, which monitors both the accuracy of the training and the accuracy of validation through 10 epochs. The training accuracy and validation accuracy are both very low at start, with the training accuracy starting at around 0.2. But, in epoch 2, both accuracies are spiking up above 0.9, showing that it is rapidly learning and predicting correctly on both the training and validation datasets. The training accuracy is always higher than the validation accuracy throughout the training process, a common behavior in machine learning models due to overfitting on the training data. Lastly, we notice that, despite these variations, both accuracies seem to settle near the end of training and ultimately, the model achieves high accuracy across both the training and validation sets, indicating that it generalizes well and performs well on unseen data.

Fig 7(d) clearly shows that dice coefficient value of each epoch with respect to procedure for segmentation, which is continuously varying with respect to DC through 10 epoch. The training and validation values are gradually upto 4 epoch and after that there is up and down in training, but for validation this gradual increase in dice coefficient as shown in the above figure.

#### 4.6.3. segmentation results of proposed model.

**Fig 8:**
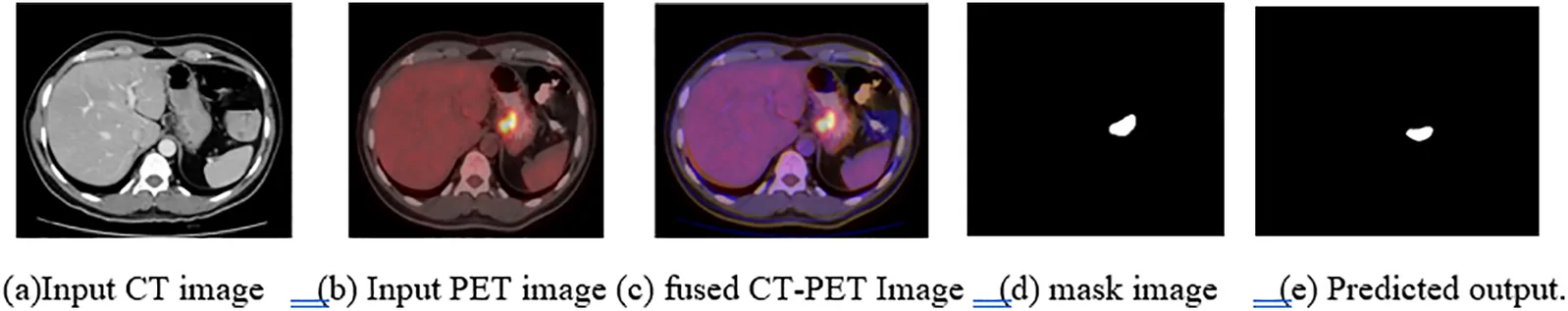
Semantic segmentation of the GIST.

Fig 8 shows the resultant segmentation region of tumour region from the fused image of the suggested model. The input fused image, which is derived from the combined CT-PET image and fed into the proposed Segformer using the MViT Encoder segmentation model, is displayed in Figure. Fig 8(a), (b) and (c) shows the resultant fused image. The mask and predicted output of proposed model are shown in Fig 8(d) & (e) respectively. respectively. the appropriate mask image and the anticipated picture obtained by semantic segmentation, respectively. It is observed that the predicted output of the proposed model is very close to mask image and results higher mIoU, accuracy and dice coefficient as shown in below.

The performance metrics, such as Dice coefficient, mean Intersection over Union (IoU) and Pixel Accuracy for the Segmented image formed by the proposed model can be seen in Table 4. This value of 93.11, 88.37 confirms that the predicted segmentation mask has an overlap of 88.37% with the ground truth mask, thus verifying that model efficiently segments the region of interest with low misclassification. The Pixel Accuracy of 97.75 also signifies the percentage of the correctly predicted pixels to the total number of pixels in the image, reassuring that the model can predict most of the pixels correctly. The high results obtained for both the precision and recall metrics signify the superiority of the segmentation model, and the accuracy of its predictions, thus, providing a strong and valid solution for medical image segmentation expert use.

**Table 4:** Performance Metrics for segmented image.

SL. No	Parameter	Predicted Value
1	Mean Inter section over union (mIoU)	88.37
2	Pixel Accuracy	97.75
3	Dice coefficient	93.11

Based on three important performance metrics—Dice coefficient, IoU (Intersection over Union) and Pixel Accuracy—Table 5 compares the suggested Segformer with MViT Encoder Model to other approaches, such as Unet with MViT, FPN with MViT, and Manet with MViT. The suggested segmenter with MViT encoder model performs well in all the metrics, like dice coefficient-93.11, an mIoU of 88.37 and a Pixel Accuracy of 97.75. With an mIoU of 84.58, Manet with MViT performs marginally better than the other models; nonetheless, the suggested model shows results that are comparable.

**Table 5:** Comparative analysis of proposed method with the existing methods.

Methodology	Dice coefficient (%)	mIoU (%)	Pixel Accuracy (%)
Unet with MViT [28]	91.43	81.96	94.56
FPN with MViT [29]	88.73	82.58	95.73
Manet with MViT [30]	90.62	85.11	93.23
Segformer with MViT Encoder Model (Proposed)	93.11	88.37	97.75

**Fig 9:**
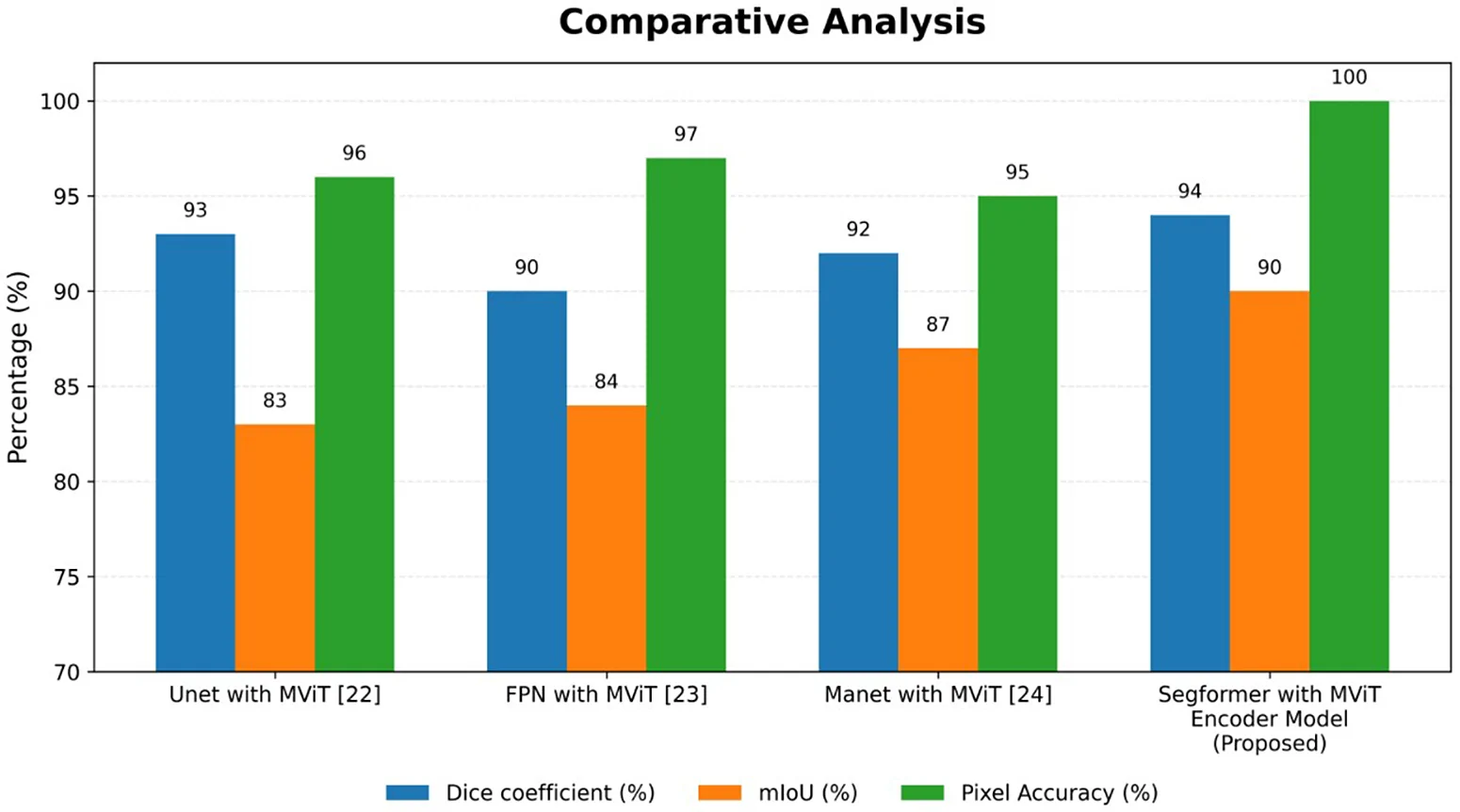
Comparative analysis of proposed model with other models.

The mIoU scores of the Unet with MViT alongside FPN with MViT are lower at 81.96 and 82.58, respectively, and the Pixel Accuracy values are marginally lower at 94.56 and 95.73. in terms of dice coefficient, the FPN with MViT and Manet with MViT results very low values of 88.73 and 90.62. The suggested Segformer with MViT Encoder Model performs better overall than the current techniques in DC, mIoU as shown in Fig 9, while retaining a high level of Pixel Accuracy, suggesting that it is useful for segmentation jobs requiring a more reliable fusion of data from both CT and PET images.

Here we also compare the performance of proposed model with existing works as shown in Table 6, it is observed that the proposed model performance is superior to other existing works in terms of Dice coefficient, IoU and Pixel Accuracy.

**Table 6:** Comparative analysis of proposed method with the existing methods.

Author & year	Dice coefficient (%)	IoU (%)	Pixel Accuracy (%)
Li, Xinyi, et al. & 2020 [31]	92.00	–	87.00
Zhuo, Minling, et al. & 2024 [32]	90.20	82.10	87.40
Pooja, K., and S. Jerritta & 2024 [33]	83.45	69.53	78.19
Ovi, Tareque Bashar, et al. & 2025 [34]	83.35	76.42	–
Feng, Qing-Chun, et al & 2026 [35]	84.70	79.20	–
Zhang, Mobei, et al & 2026 [36]	88.40	84.21	81.40
Proposed work	93.11	88.37	97.75

## 5. Conclusion

By merging CT and PET images to create a complete image of the Tumour, the proposed model successfully improves GIST detection and segmentation. The significance of the research was that successfully proposed two stage deep learning models for accurate segmentation of fused image. The DIF-Net model, an advanced model integrated the Segformer with MViT Encoder architecture, to achieve the CT-PET image fusion and segmentation. The key results show that the proposed model achieves better segmentation accuracy and fusion quality compared with existing methods. The DIF-Net model has strong ability to capture the high-resolution anatomical details and functional information from CT and PET modalities, which encourages more accurate segmentation performance. It was demonstrated that using MViT Encoder in the Segformer is especially effective in overcoming the challenges of multi-modal data, resulting in efficient and accurate segmentation. The proposed results DC of 93.11, 88.37 mean IoU and 97.75-pixel accuracy, which are a substantial improvement against other methods.

## 6. Limitations and future scope

Here, we highlight a few limitations even though the suggested SegFormer-Mix Vision Transformer-based system shows encouraging performance for GIST segmentation in fused CT-PET data. First, the model’s capacity to generalize across various clinical contexts and imaging methods is impacted by the very small dataset used for evaluation. Second, the proposed work has not been validated on multimodal imaging datasets or other tumor types; it solely focuses on GIST tumor segmentation.

Future research will focus on validating the proposed model using larger multi-centre datasets to improve robustness and generalizability. Additionally, the framework can be extended to segment other tumours from multimodal medical images. Furthermore, integrating advanced multimodal fusion strategies and hybrid CNN-transformer architectures may further improve segmentation accuracy and diagnostic support.
